# Mupirocin blocks imiquimod-induced psoriasis-like skin lesion by inhibiting epidermal isoleucyl-tRNA synthetase

**DOI:** 10.1186/s12964-022-00995-0

**Published:** 2022-11-22

**Authors:** Bing-Xi Yan, Xue-Yan Chen, Zhao-Yuan Wang, Ying-Zhe Cui, Lilla Landeck, Ni-Chang Fu, Xing-Yu Yang, Fan Xu, Yuan Zhou, Jia-Qi Chen, Xiao-Yong Man

**Affiliations:** 1 Department of Dermatology, Second Affiliated Hospital, Zhejiang University School of Medicine, 88Th Jiefang Road, Hangzhou, 310009 China; 2 Ernst Von Bergmann General Hospital, Teaching Hospital of Charité, University Medicine Berlin–Humboldt University Berlin, Potsdam, Germany

**Keywords:** Mupirocin, Imiquimod, IARS, Psoriasis

## Abstract

**Background:**

The Isoleucyl-tRNA synthetase (IARS) catalyzes isoleucine to the corresponding tRNA, maintaining the accuracy of gene translation. Its role in psoriasis has been not investigated so far. In this study, we aimed to investigate the mechanisms underlying the efficacy of IARS inhibitor, mupirocin, treatment for psoriasis.

**Methods:**

The expression of IARS was determined by immunofluorescence, Western blot and qRT-PCR in normal healthy control- and psoriatic human skin. An imiquimod (IMQ) -induced psoriasis-like skin disease model was used to study the phenotypes changed by an IARS inhibitor, mupirocin (MUP). Endotypes were analyzed by RNA-seq, R&D Luminex multi-factor technique, ELISA, immunofluorescence and flow cytometry. Additionally, the effect of MUP on epidermal keratinocytes (KCs) were conducted in-vitro in primary cultured human KCs.

**Results:**

We found the expression of IARS was higher in psoriatic skin than in healthy controls. In IMQ-induced psoriasis-like C57BL/6 J mouse model, MUP reversed IMQ-induced keratinocytes proliferation, expression of inflammatory cytokines and infiltration of immune cells. Furthermore, in cultured human keratinocytes, MUP inhibited proliferation, but promoted apoptosis, which may be related with STAT3 signaling pathway.

**Conclusion:**

Our finding of blocking the infiltration of immune cells by inhibiting the formation of IARS, could be one mechanism to explain the effect of MUP in the treatment of psoriasis. Developing strategies targeting suppression IARS should open new perspectives for the treatment of psoriasis.

**Graphical Abstract:**

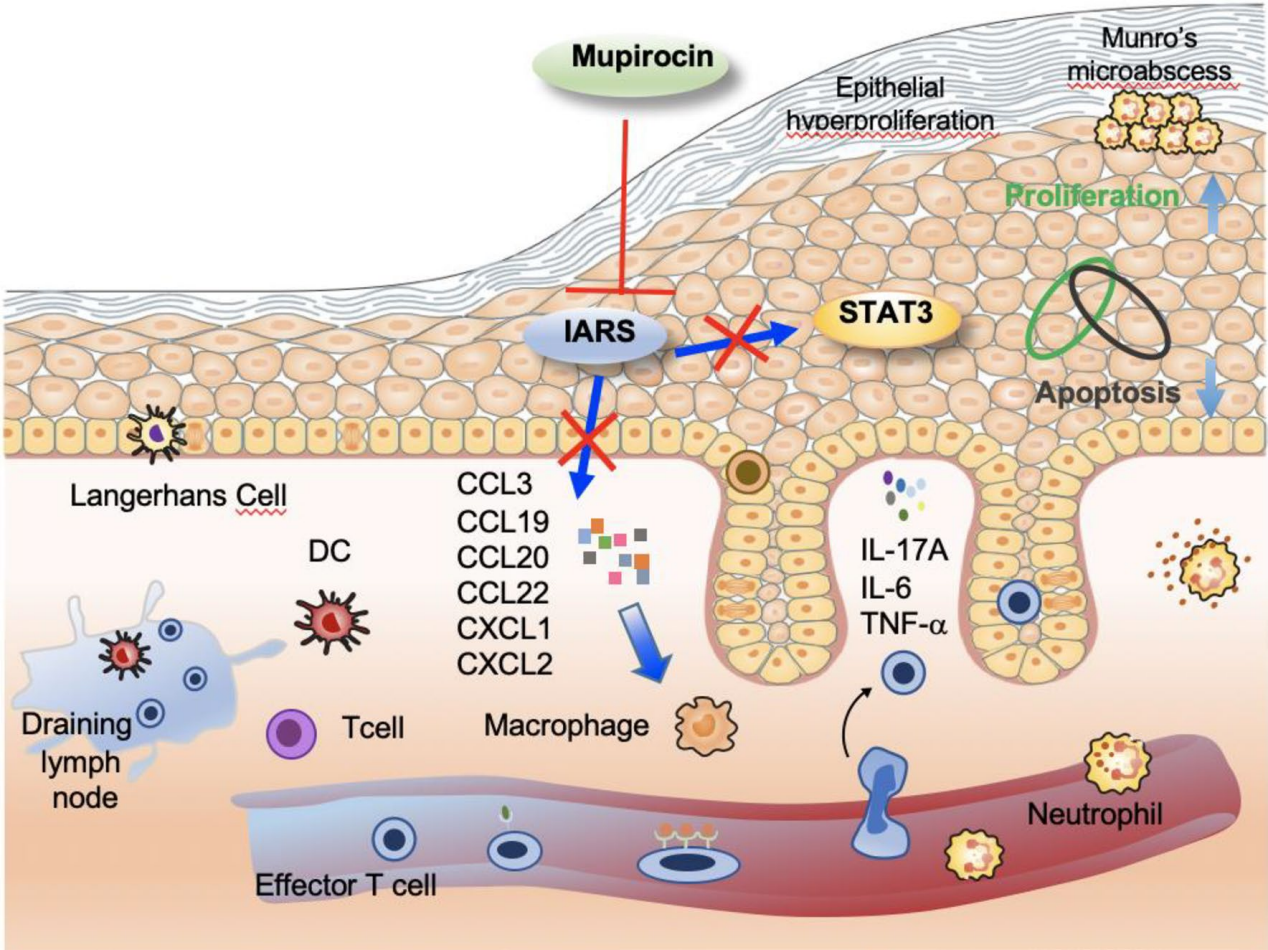

**Video Abstract**

**Supplementary Information:**

The online version contains supplementary material available at 10.1186/s12964-022-00995-0.

## Introduction

Psoriasis is a common immune-mediated chronic inflammatory disease that affects 2%-3% of the general population in developed countries [1–5]. Although the pathogenesis of psoriasis is still insufficiently understood, it is believed to arise from an interplay between genetic susceptibility and triggering environmental factors (such as infections, skin injury, and stress), both are likely to be of significant importance in the development of psoriasis [6].

Both keratinocytes and immune cells contribute to the pathogenesis of psoriasis [7], and IL-23 and IL-17A were identified as key drivers of this immune process. Blocking IL-23 or IL-17A shows highly clinical efficacy in the treatment of psoriasis [8–12]. Moreover, microbial pathogens have been identified to be highly co-evolved relationship with the immune system at barrier tissues, especially in the skin [13]. Previous studies showed a significantly increased *colonization of Staphylococcus*, especially *Staphylococcus aureus,* species in psoriatic skin lesions [14, 15]. Therefore, the interaction between infection and the immune system may play a critical important role in the pathogenesis of psoriasis.

Mupirocin (MUP) is a topical antibiotic agent against skin infections caused by *Staphylococcus aureus*. As we known, it is not used as a standard treatment of psoriasis, but in case of colonization with bacteria [16]. Specifically, MUP targets the aminoacylation site of bacterial Isoleucyl-tRNA synthetase (IARS) [17, 18]. IARS is a member of aminoacyl-tRNA synthetases (ARSs) that ligate amino acids to their corresponding tRNAs in protein synthesis [19]. In the last few years, ARSs have been considered as a potential drug for the treatment of a few diseases, such as ovarian, renal, cervical and breast cancer [20]. In addition, IARS has been reported as a target to prevent abdominal aortic aneurysm by down-regulating the activity of the p38 MAPK pathway [21, 22].

Here, through analysing the GEO database (GSE13355) [23], we found that mRNA expression of IARS was significantly increased in psoriatic lesional skin compared to non-lesional—or normal skin. Based on this finding, we ought to investigate the role of IARS in the pathogenesis of psoriasis. Furthermore, we found that MUP inhibited the process of IMQ-induced psoriasis-like dermatitis in vivo by inhibiting the activity of IL-17A signaling.

## Materials and methods

### Isolation and culture of NHEKs and PLEKs

NHEKs and PLEKs were established from skin biopsy samples that were incubated with dispase, as described earlier [24]. Briefly, detached keratinocytes without contamination were seeded onto flasks at a density of 5000 cells/cm^2^ and maintained in Epilife Medium (Gibco, USA) containing human keratinocyte growth supplement (Gibco, USA) with media refreshed every 48–72 h. The NHEKs and PLEKs were cultured at 37 °C and 5% CO_2_ in a humid atmosphere.

### Western blot

Western blot analysis was performed as described earlier [24]. Primary antibodies used were anti-IARS (1:1000, abcam, 31533), anti-STAT3 (1:1000, Cell Signaling Technology, #4180), anti-pSTAT3 (1:1000,9145S,CST), anti-β-actin (1:10000, 4970S, CST), anti-Bax (1:1000, Santa Cruz, sc-20067), anti-Caspase 3 (1:1000, Proteintech, 19677-1-AP). Secondary antibodies used in immunoblotting studies were HRP-conjugated (1:5000, Jackson Immuno Research). Signals were revealed by enhanced chemiluminescence kit (Millipore).

### Quantitative real-time PCR

Total RNA was isolated from keratinocytes of healthy donors serving as controls and psoriatic patients by using TRIzol reagent (Invitrogen, Carlsbad, CA, USA). qRT-PCR was done as described earlier [25]. CT values were analyzed by qBase Plus 2 software (Biogazelle, Zwijnaarde, Belgium).

### Enzyme-linked immunosorbent assay (ELISA)

Epidermis protein of sacrificed modeling mice were collected and stored at -80 °C and subjected to a single freeze–thaw cycle. Mouse IL-17A kits from BOSTER (Wu Han, China) were used. ELISA assay was performed according to the manufacturer’s instructions.

### Cell apoptosis assays

The cell apoptosis assays were performed as described earlier [26]. Cells stained with an Annexin V-FITC/PI apoptosis kit (BD Biosciences, Franklin Lakes, NJ, USA) according to the manufacturer’s instructions, and analyzed using a flow cytometer. FlowJo software (Version 7.6.1, Treestar, Ashland, OR, USA) was used for subsequent analysis.

### Cell viability assay

The CCK-8 assay was performed as we described earlier [27]. When running the assay, reagents from a CCK-8 reagent-based kit (Promega, USA) absorbance at 450 nm was measured using a plate reader (BioTek, USA). Cell proliferation was detected using the incorporation of 5-ethynyl-29-deoxyuridine (EdU) with the EdU Cell Proliferation Assay Kit (Invitrogen, USA) according to the manufacturer’s protocol. The EdU proliferation assay was performed as described earlier [26]. The proportion of cells that incorporated EdU was determined using by fluorescence microscopy (Leica, German).

### Mouse

Eight-week-old female C57BL/6 J mice with an average bodyweight of approximately 20 g were bought from the Shanghai Laboratory Animal Co. Ltd (Shanghai, China). Mice were fed with normal forage and provided with water ad libitum in the animal experimental center of the Second Affiliated Hospital, Zhejiang University School of Medicine.

### Mouse model of imiquimod (IMQ)-induced psoriasiform skin inflammation

Mice were numbered and randomly assigned to six groups in which the corresponding forages were given for 1 week. Then, an area of 3 cm × 5 cm was shaved from the back of all mice and treated with a daily topical dose of 12.5 μg of imiquimod cream (5%) (MED.SHINE, China) for six consecutive days. 0.6 g of Mupirocin (2%) (SK&F) or vehicle as a control was administered to C57BL/6 J mice after the topical use of imiquimod. The detailed administration of the six groups is described in the result section. PASI scores were performed as we described earlier [26]. Mice were sacrificed by dislocation of the neck.

### Flow cytometry analysis

For each staining, cells (1 × 10^6^cells/ml) were fluorescently labeled after incubation in a dark room for 30 min at room temperature. Monoclonal antibodies are listed in the Additional file 1: Table E2 in this article. All of the samples were detected on a CytoFLEX LX (Beckman Coulter) and analyzed with Kaluza analysis software (Beckman Coulter).

### Immunohistochemical- & Immunofluorescence staining analyses

The back skin of mice was fixed in 10% formalin and embedded in paraffin. An immunohistochemical analysis was performed using the standard ABC-peroxidase Kit (Vector, Burlingame, Ontario, CA, USA) as suggested by the manufacturer. Affinity-purified biotinylated anti-rabbit and anti-mouse IgG was purchased from Vector Lab (Burlingame, Ontario, CA, USA). Other antibodies used in this study including anti-cytokeratin14 (1:1000, abcam, 181,595), anti-Ki67 (1:1000, abcam, 16,667). Immunofluorescence was performed according to our previous published work [24, 27].The following antibodies were used: anti-IARS (1:200, abcam, 31,533), anti-CD4 (1:1000, abcam, 183,685), anti-CD8(1:100, R&D, MAB116), anti-Ly6G (1:1000, abcam, 238,132). Secondary antibodies labeled with Cy3 (Red) (1:5000, Jackson Immuno Research) were used.

### Statistical analysis

Prism 8 software (GraphPad, SanDiego, CA, USA) was used to perform the statistical analysis. Student’s t-test and one-way ANOVA test were used depending on the experimental conditions. All the data are presented as mean ± SEM (Standard Error of Mean), and *p* < 0.05 was considered statistically significant.

### Ethics

The Animal Care and Use Committee of Second Affiliated Hospital, Zhejiang University School of Medicine approved all animal procedures of this study. Human skin was obtained from 3 healthy adult donors and 6 psoriasis patients after written informed consent was obtained. The protocol was approved by the Institutional Review Board at Second Affiliated Hospital, Zhejiang University School of Medicine.

## Results

### IARS was highly expressed in psoriatic epidermis

**Fig. 1:**
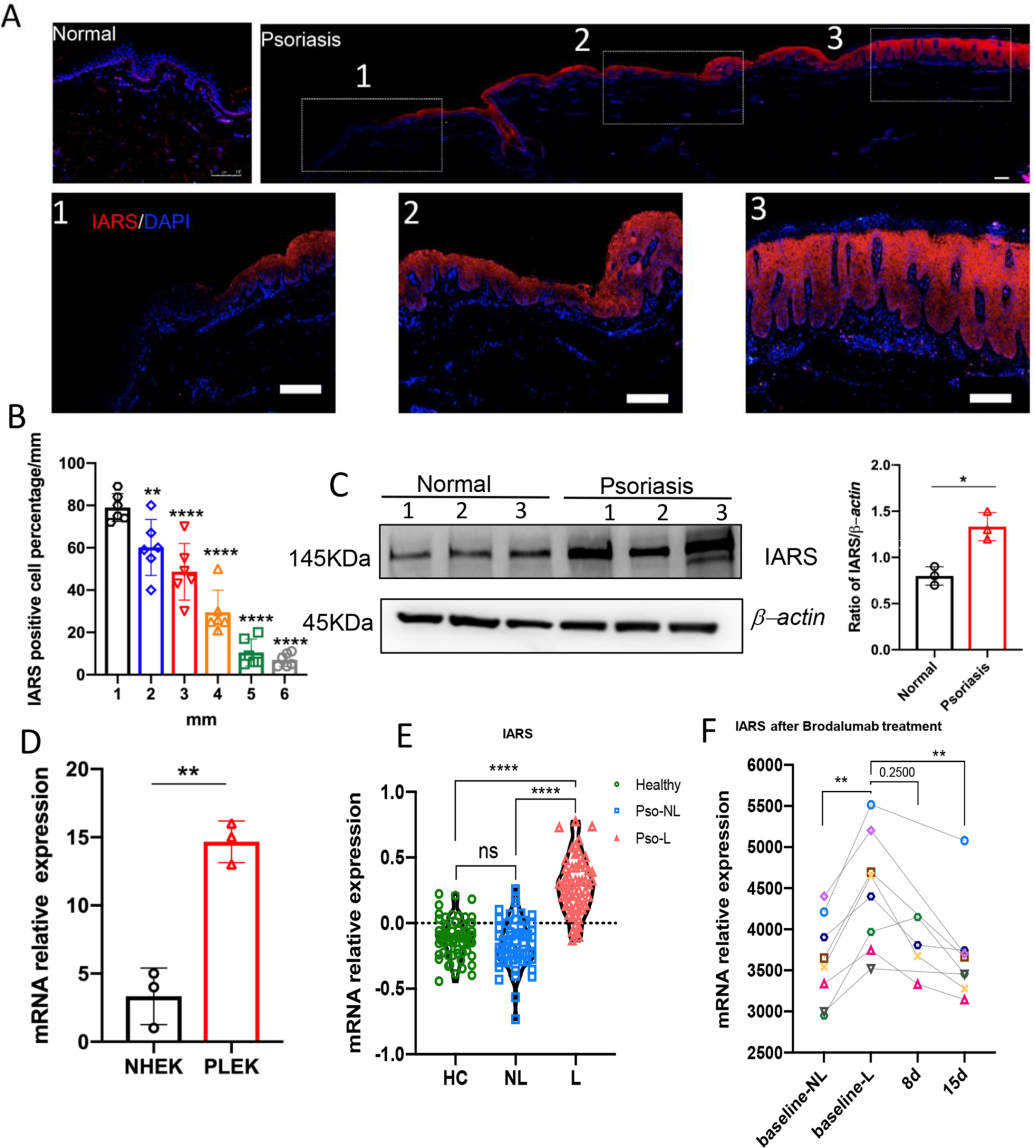
Expression profile of IARS in human normal skin, psoriasis patient skin, NHEKs and PLEKs. **A** Representative immunofluorescence images of IARS in the skin of normal person (left) and psoriatic patient (right). Scale bar = 100 μm. The 1-2-3 of the dashed boxes represent the enlarged non-lesional skin, peri-lesional skin and lesional skin respectively. **B** Quantification analysis of immunofluorescence staining of IARS positive cell percentage for psoriasis patient of different distance (n = 6 ± SEM), **P < 0.01 versus 1 mm, ****P < 0.0001 versus 1 mm. **C** The western blot analysis was performed to measure the expression of IARS from normal and psoriatic epidermis (n = 3 ± SEM) *P < 0.05. **D** Relative mRNA expression levels in NHEKs and PLEKs were determined by qRT-PCR: IARS (n = 3 ± SEM) **P < 0.01. **E** Microarray detected the relative expression of IARS in human psoriatic lesional skin (L, n = 58), non-lesional skin (NL, n = 58) and healthy controls (HC, n = 64). Mann Whitney test was used in this analysis (****P < 0.0001 vs. lesional). **F** The expression of IARS in the skin of psoriasis patients were detected by Affymetrix Human Genome U133 Plus 2.0 Array, before and 8 days/15 days after anti-IL-17RA (brodalumab, 350 mg) treatment. Each color dot represents individual patient (n = 8), and Wilcoxon matched-pairs signed rank test was used in this analysis (**P < 0.01 vs. baseline-lesional)

To investigate the expression profile of IARS in healthy donor epidermis and psoriatic epidermis, immunofluorescence (IF) staining against IARS was performed in skin biopsies obtained from healthy donors (n = 3) and psoriasis patients (n = 6). Compared with normal epidermis, IARS has significantly been upregulated in psoriatic epidermis (Fig. 1A, B). IARS was expressed in all epidermal layers in lesional skin. In perilesional skin, it was only expressed in the suprabasal layers of the epidermis as shown in Fig. 1a. We calculated the percentage of IARS positive cells at 1 mm, 2 mm, 3 mm, 4 mm, 5 mm, and 6 mm from the lesional area, which represented decreasing epidermal thickness. As shown in Fig. 1b, the percentage of IARS positive cells gradually decreased parallel to reduced thickness. In order to further confirm the up regulation of IARS in psoriatic lesions, we detected its protein level in normal and psoriatic epidermis by Western blot. Compared with normal epidermis, the protein level of IARS was increased in the psoriatic epidermis (Fig. 1C). Meanwhile, normal human epidermal keratinocytes (NHEKs) and psoriasis lesional epidermal keratinocytes (PLEKs) were used to detect the expression of IARS at a transcriptional level by qRT-PCR. Compared with NHEKs, the mRNA level of IARS was significantly up regulated in PLEKs (Fig. 1D). We searched the GEO database, were two published datasets GSE13355 [23] and GSE53552 [28] showed that IARS expression was significantly increased in the lesional skin of psoriasis patients, which was reversed after blocking IL-17 signaling by brodalumab (Fig. 1E, F).

### IMQ-induced psoriasis-like skin inflammation in mice was alleviated by topical mupirocin

**Fig. 2:**
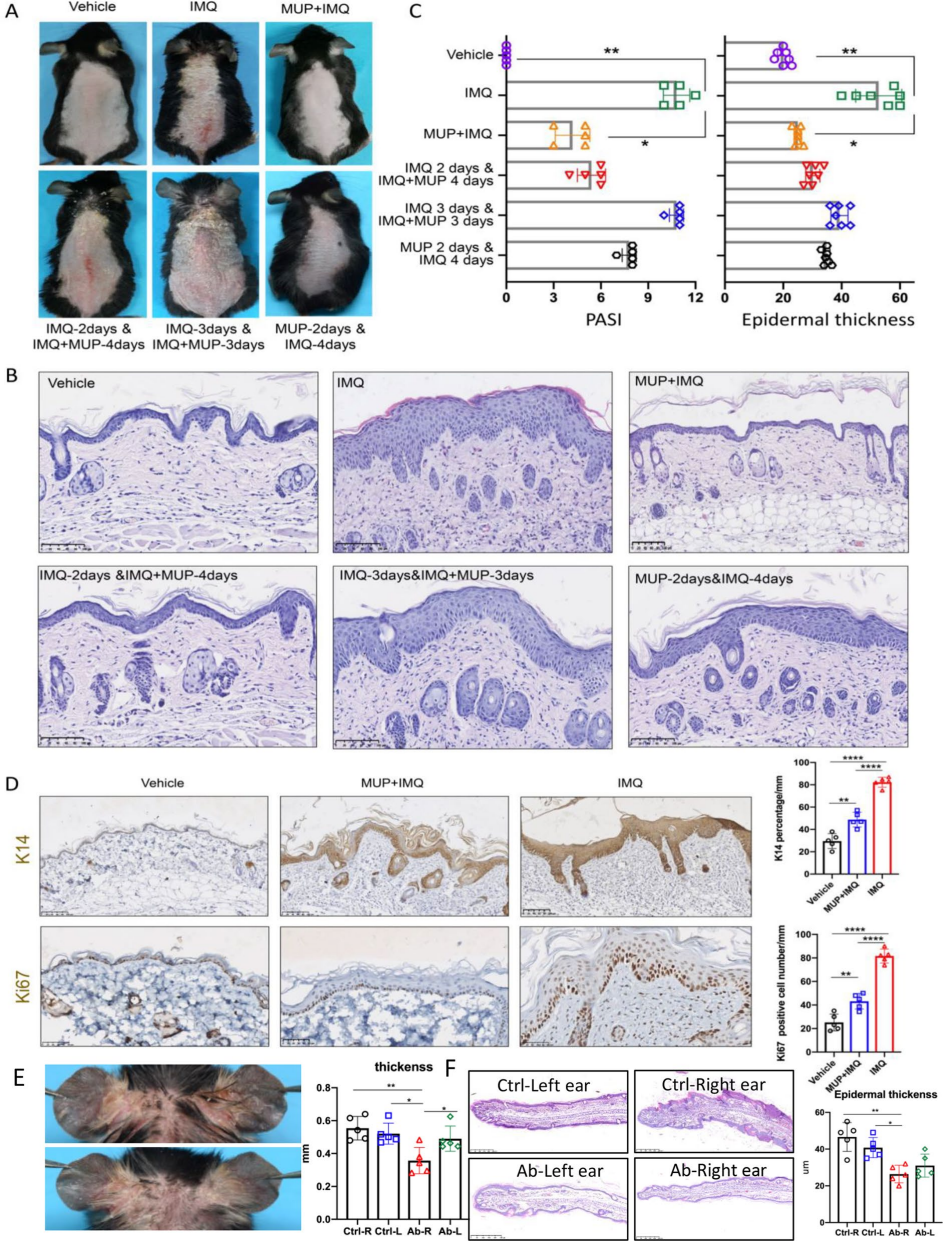
Mupirocin administration attenuated IMQ-treated mice. **A** Representative clinical presentations of vehicle-treated mice, IMQ-treated mice and MUP + IMQ-treated mice (top line from left to right), IMQ-2 days & IMQ + MUP-4 days-treated mice, IMQ-3 days & IMQ + MUP-3 days-treated mice and MUP-2 days & IMQ-4 days-treated mice (bottom line from left to right). **B** Representative H&E images of dorsal skin from different six treated mice described as (A). Scale bar = 100 μm. **C** PASI score (left) and epidermal thickness (right) was performed of day 6 from the six groups described as (**A**) (n = 5 ± SEM). *P < 0.05, **P < 0.01 vs. IMQ. **D** Representatives IHC images and quantification analysis of keratin14 (top) and Ki67 (bottom). Scale bar = 100 μm. (n = 5 ± SEM). ** *P* < 0.01 vs. vehicle, ****P < 0.0001 vs. IMQ). **E** Representative clinical presentations of IMQ-treated mice of the 0.9% NaCl-injection group (right ear) (top) and anti-IARS-injection group (10ul, abcam,ab315333, from 2nd to 5th day) (right ear) (bottom). Quantification analysis of ear thickness of the 0.9% NaCl-injection group and anti-IARS-injection group. (n = 5 ± SEM). * *P* < 0.05** *P* < 0.01. **F** Representative H&E images(left) and the average epidermal thickness(right) of ear skin from IMQ-treated different four mice groups described as (**E**). Scale bar = 100 μm. (n = 5 ± SEM). *P < 0.05, **P < 0.01

Since IARS was highly expressed in psoriatic epidermis, we used the inhibitor of IARS, mupirocin (MUP), to further explore the role of IARS in psoriasis. To assess whether topical mupirocin could relief IMQ-induced psoriasis-like skin inflammation in vivo, the experimental mice were treated with 6 different methods: 1) blank control (vehicle-treated mice); 2) topical application of IMQ for 6 consecutive days (IMQ-treated mice); 3) topical application of MUP and IMQ for 6 consecutive days (MUP + IMQ-treated mice); 4) topical application of IMQ for 6 consecutive days, accompanied by MUP at the 3^rd^ to 6^th^ day (IMQ-6 days&MUP-4 days-treated mice); 5) topical application of IMQ for 6 consecutive days,, with MUP application at the 4^th^ to 6^th^ day(IMQ-6 days&MUP-3 days-treated mice); 6)topical application of MUP for 2 consecutive days, followed by IMQ for another 4 days (MUP-2 days&IMQ-4 days-treated mice)(Fig. 2a). Compared with the vehicle-treated mice, typical psoriasis-like skin lesions were shown in the IMQ-treated mice. However, these lesions were significantly relieved in the MUP + IMQ-treated mice with reduced scales and erythema. In group 4 and 5, we found that decreased application of mupirocin resulted in exacerbation of psoriasis-like lesions. In addition, mice developed typical psoriasis-like skin lesions even when mupirocin was applied 2 days in advance. The PASI scores at the 6^th^ day of MUP + IMQ-treated mice were significantly lower than that of the IMQ-treated mice (n = 5, *P < 0.05, **P < 0.01) (Fig. 2C). Then, H&E (hematoxylin-eosin) staining was performed to evaluate epidermal thickness and histopathological changes. Compared with the IMQ-treated mice, the MUP + IMQ-treated mice showed an obvious decrease in the severity of psoriasiform skin changes (Fig. 2B). Moreover, the epidermal thickness was significantly reduced (n = 5, *P < 0.05, **P < 0.01) (Fig. 2C). In addition, keratin 14(K14) and Ki67, as cell proliferation-related indicators, were detected using immunohistochemistry. Upon IMQ induction, K14-positive cells were located in all layers of the epidermis. However, the percentage of K14-positive cells was significantly decreased after application of mupirocin (Fig. 2D). Similar results of Ki67 positive cells were observed between these two groups (Fig. 2D). Furthermore, the ear thickness was significantly decreased after IARS-antibody injection (n = 5, *P < 0.05, **P < 0.01) (Fig. 2E, F). Taken together, the phenotypic and histological features of IMQ-induced psoriasis-like skin inflammation were improved by topical mupirocin and IARS-antibody injection treatment.

### Mupirocin inhibited epidermal IARS in IMQ-induced psoriasis-like mouse model with decreased expression of cytokines and chemokines in the IL-17 signaling pathway

**Fig. 3:**
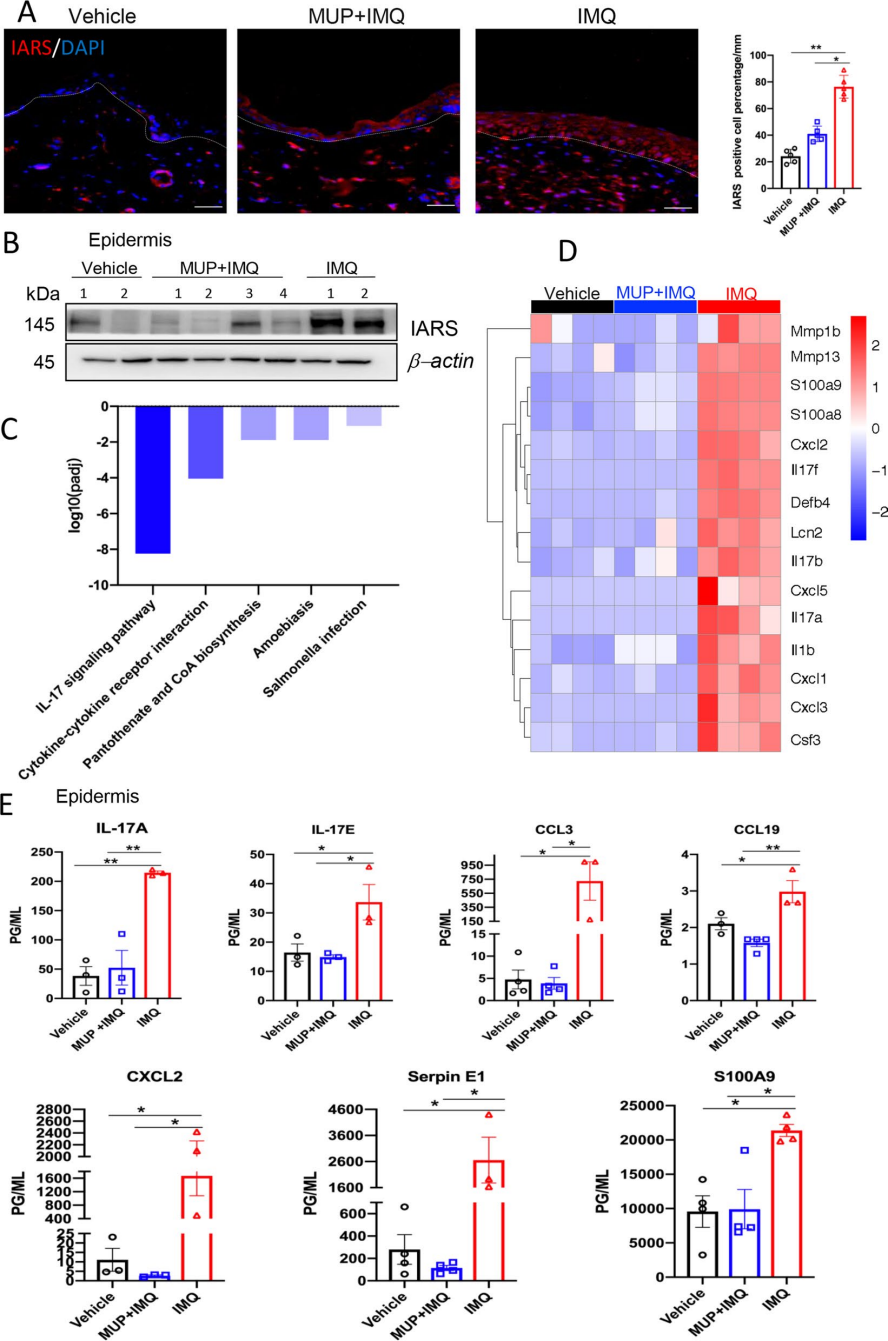
Mupirocin inhibited the expression of IARS in the epidermis of IMQ-induced mice associated with IL-17 signaling pathway. **A** Representative immunofluorescence images (left) and quantification analysis (right) of IARS on the skin of the vehicle-treated, MUP + IMQ-treated and IMQ-treated mice. Scale Bar = 100 μm. (n = 5 ± SEM). * *P* < 0.05, ** *P* < 0.01 versus vehicle. **B** A western blot analysis was performed to measure the expression of IARS of epidermis from the three treated mice. *β-actin* was used as loading control. **C** Based on the RNA-seq analysis of mouse epidermis, KEGG was used to analyze the signal pathways of different genes in the epidermis of the MUP + IMQ-treated and IMQ-treated mice. **D** Based on the results of RNA-seq, analyze the differential gene expression in the epidermis of the three treated mice (n = 4) including: Mmp1b, Mmp13, S100a9, S100a8, Cxcl2, Il17f, Defb4, Lcn2,Il17b,Cxcl5,Il17a,Il1b,Cxcl1,Cxcl3 and Csf3. **E** Biolegend Elisa detection kit detects the expression of IL-17A in the epidermis of three treated mice; R&D Luminex Multi-Analyte Assay detects the expression of IL-17E, CCL3, CCL19, CXCL2, Serpin E1, and S100A9 protein in the epidermis of three treated mice. (n = 3 ± SEM), *P < 0.05, ** *P* < 0.01

To determine, whether mupirocin is able to decrease epidermal IARS and inflammatory cytokine- and chemokine expression, we compared the expression of IARS and some cytokine- and chemokine expression levels among vehicle-treated mice, MUP + IMQ treated mice and IMQ-treated mice. As shown in Fig. 3A, IMQ-treated mice showed full-layer IARS expression in the epidermis, which was consistent with psoriatic lesions of patients. Furthermore, when treated with MUP and IMQ, IARS protein level was significantly decreased (Fig. 3A). Immunoblotting results showed similar expression differences among these 3 groups (Fig. 3B). To look for the possible signaling pathway, RNA sequencing (RNA-Seq) was performed with mouse epidermis. Differentially expressed genes (DEGs) were discovered by DESeq with the threshold of |log2 fold change|> 2 and adjusted *P* value < 0.05. Then, all down-regulated DEGs between MUP + IMQ-treated and IMQ-treated were analyzed by Kyoto Encyclopedia of Genes and Genomes (KEGG). These DEGs were statistically enriched in IL-17 signaling pathways. Among them, the top 5 signaling pathways were the IL-17 signaling pathway, the cytokine-cytokine receptor interaction, pantothenate and CoA biosynthesis, amoebiasis and salmonella infection (Fig. 3C). It is well known that the IL-23/IL-17 immune axis is a key driver in the development of psoriasis. Here, 15 DEGs were enriched in the IL-17 signaling pathway, including IL-17 family (IL-17A, IL-17B and IL-17F), chemokines (CXCL1, CXCL2 and CXCL3), and S100 protein family members (S100A8 and S100A9), etc. (Fig. 3D). RNA-Seq results of mouse epidermis suggested that many cytokines (IL-17 family, IL-1β) and chemokines (CXCL1, CXCL2 and CXCL3) were reduced in MUP + IMQ-treated mice. To further analyze their expression at the protein level, Luminex Multi-Analyte Assay was applied to mouse epidermis. As shown in Fig. 3E, chemokines (CXCL2), and S100 Protein family (S100A9) were highly expressed in the epidermis of IMQ-treated mice compared with vehicle-treated and MUP + IMQ-treated mice, which was consistent with the RNA-Seq results. IL-17 family cytokine (IL-17E), chemokines (CCL3, CCL19), and Serpin E1 were down regulated in MUP + IMQ-treated mice. Among them, the concentration of IL-17A was detected by ELISA detection kit for epidermal protein. Results showed that the IL17A concentration in the epidermis of IMQ-treated mice was significantly higher than that in the vehicle-treated- and MUP + IMQ-treated mice as shown in Fig. 3D. All of these findings were consistent with the transcriptome expression.

### Mupirocin decreased the inflammatory cell infiltration in IMQ-induced mouse model

**Fig. 4:**
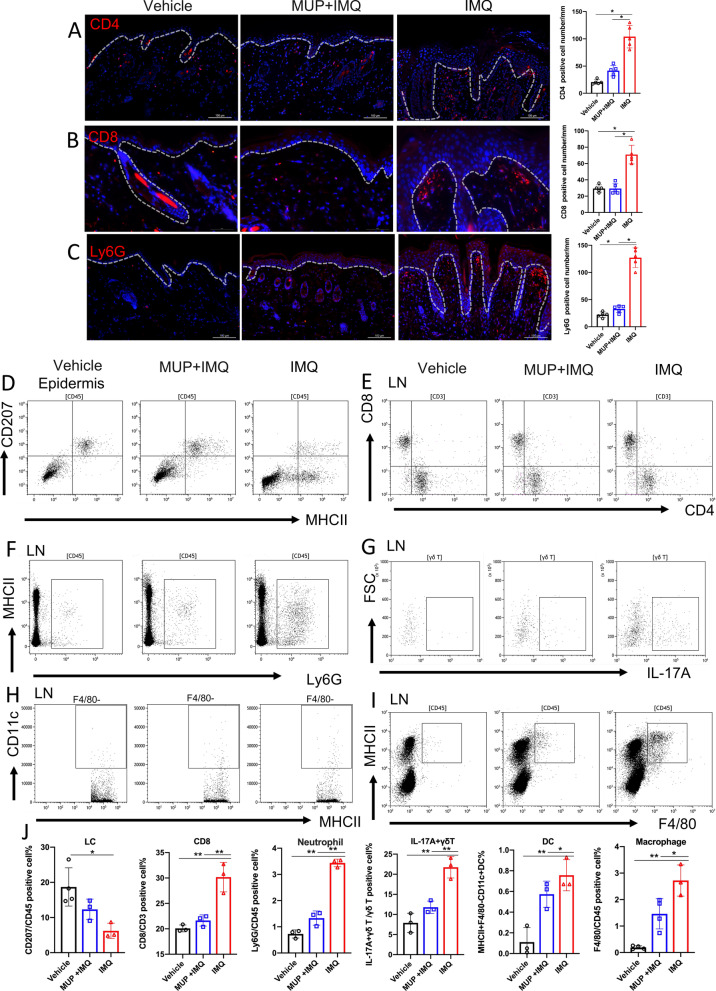
Mupirocin altered immunocytes compositions in IMQ-treated mice. Representative immunofluorescence images (right) and quantification analysis (left) of CD4(**A**), CD8(**B**), and Ly6G(**C**) on the skin of the vehicle-treated, MUP + IMQ-treated and IMQ-treated mice. Scale Bar in (**A**) and (**C**) is 100 μm. Scale Bar in (**B**) is 50 μm. (n = 5 ± SEM), *P < 0.05 versus vehicle. Representative flow results of MHCII^+^CD207^+^cell (**D**) and percentage analysis (**J**) in the epidermis. Representative flow results of CD3^+^CD8^+^T cell (**E**), Ly6G^+^ neutrophils(**F**), IL-17^+^ γδT cell (**G**), MHCII^+^F4/80^−^CD11c^+^ DC cell(**H**)and MHCII^+^F4/80^+^ macrophage (**I**) and quantification analysis (**J**) in the LN. (n = 3 ± SEM). *P < 0.05, ** *P* < 0.01 versus IMQ. Flow cytometry gating strategies are showed in the Additional file 2

The infiltration of inflammatory cells in skin and lymph nodes (LN) was analyzed by IF, IHC and flow cytometry. In the IMQ-treated mice group, the number of CD4^+^ T cells and CD8^+^ T cells were significantly increased in the skin and draining lymph nodes compared with vehicle-treated mice, which could be reversed by MUP treatment at the same time (Fig. 4A, B, E). As “Munro” micro abscess is a typical pathological feature of psoriasis [29], we studied the number of neutrophils in the skin and the LN. Neutrophil numbers increased in the IMQ-induced psoriasis-like skin inflammation, but decreased significantly after MUP application (Fig. 4C). Similar results were observed in LN (Fig. 4F, J). Flow cytometry results of the epidermis showed that compared with IMQ-treated group, the percentage of LCs in the vehicle-treated and MUP + IMQ-treated mice were increased (*P* < 0.05, Fig. 4D–J). Through the participation of skin draining LN, the initial immune response will generate a self-sustaining inflammatory cycle to maintain inflammation. The flow cytometry of LN showed similar results. The proportion of CD3^+^CD4^+^, CD3^+^CD8^+^ cells (Fig. 4E), neutrophils (Fig. 4F), IL-17A secreting γδT cells (Fig. 4G), dendritic cells (Fig. 4H), macrophages (Fig. 4I) and in the IMQ-treated mice were significantly higher than that in the vehicle-treated and MUP + IMQ-treated mice. (*P* < 0.05 and *P* < 0.01, Fig. 4J).

### Mupirocin inhibited the proliferation of PLEKs and promoted their apoptosis

**Fig. 5:**
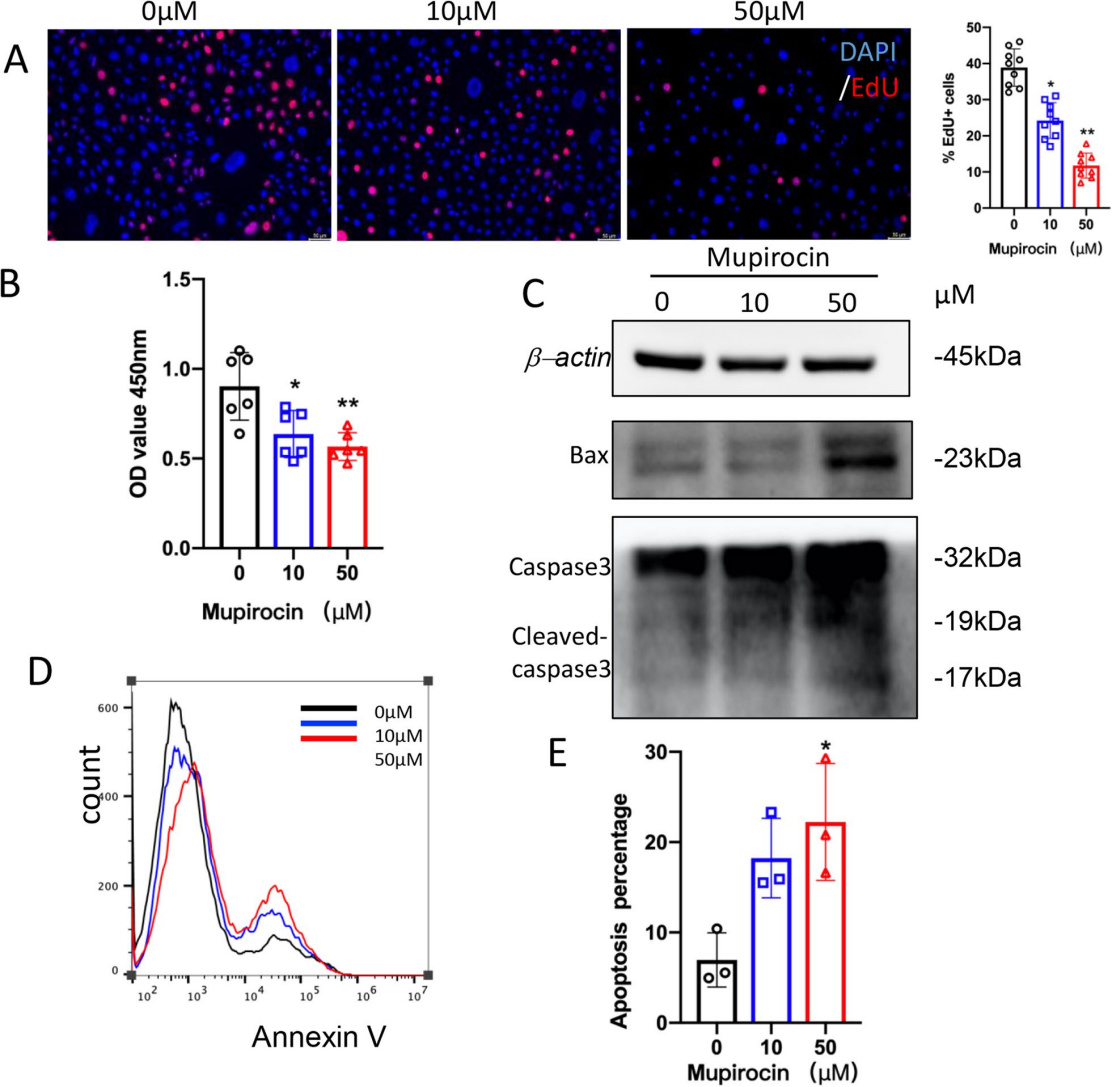
Mupirocin inhibited cell proliferation while promoted apoptosis in PLEKs. PLEKs were treated with various concentrations of mupirocin. **A** Cell proliferation ability in PLEKs treated for 48 h was evaluated using EdU staining (right). Red is EdU-555 staining. Scale Bar = 50 μm. The percentage viability (live cell count/total cell count) was calculated and expressed as mean ± SEM in five representative fields for each group (left). *P < 0.05, **P < 0.01 versus 0 μM. **B** Comparison of cell proliferation in PLEKs treated for 48 h by CCK-8 assay. (n = 6 ± SEM). *P < 0.05, **P < 0.01 versus 0 μM. **C** Western blotting analysis of Bax, Caspase3 and cleaved Caspase-3 protein expression in PLEKs treated for 24 h. *β*-actin was used as the loading control. **D** Apoptotic cell-death was measured by annexin V-FITC/PI staining in PLEKs treated for 48 h. **E** The graph represents the mean ± SEM of the percent of apoptotic cells in the flow cytometry results. (n = 3). *P < 0.05, versus 0 μM

As keratinocytes play an important role in the pathogenesis of psoriasis, we further explored the effect of IARS on keratinocytes. PLEKs were treated with several concentrations of mupirocin (0 μM, 10 μM and 50 μM) for 48 h. Then the effects on the proliferation and apoptosis activities of keratinocytes were studied. First, the cell proliferation of the mupirocin-treated keratinocytes was determined by EdU assay. Compared with the control group, the number of EdU-positive cells were significantly decreased after mupirocin treatment in a dose-dependent manner (*P* < 0.05; *P* < 0.01; Fig. 5A). Moreover, the cell density of mupirocin-treated PLEKs was also markedly reduced (*P* < 0.05; *P* < 0.01; Fig. 5B). Also, we found that mupirocin could induce the expression of Bax and cleaved-caspase3 in keratinocytes, especially at the concentration of 50 μM (Fig. 5C). In addition, flow cytometry with Annexin V-FITC/PI double staining was performed to determine the percentage of apoptotic cells. When the concentration of mupirocin reached 50 μM, a significant increase in the percentage of apoptotic cells in the mupirocin-treated PLEKs (*P* < 0.05; Fig. 5D, E) was detected. Briefly, the results above suggest that mupirocin could inhibit the proliferation of PLEKs and promote their apoptosis.

### Regulation of IARS in PLEKs and NHEKs

**Fig. 6:**
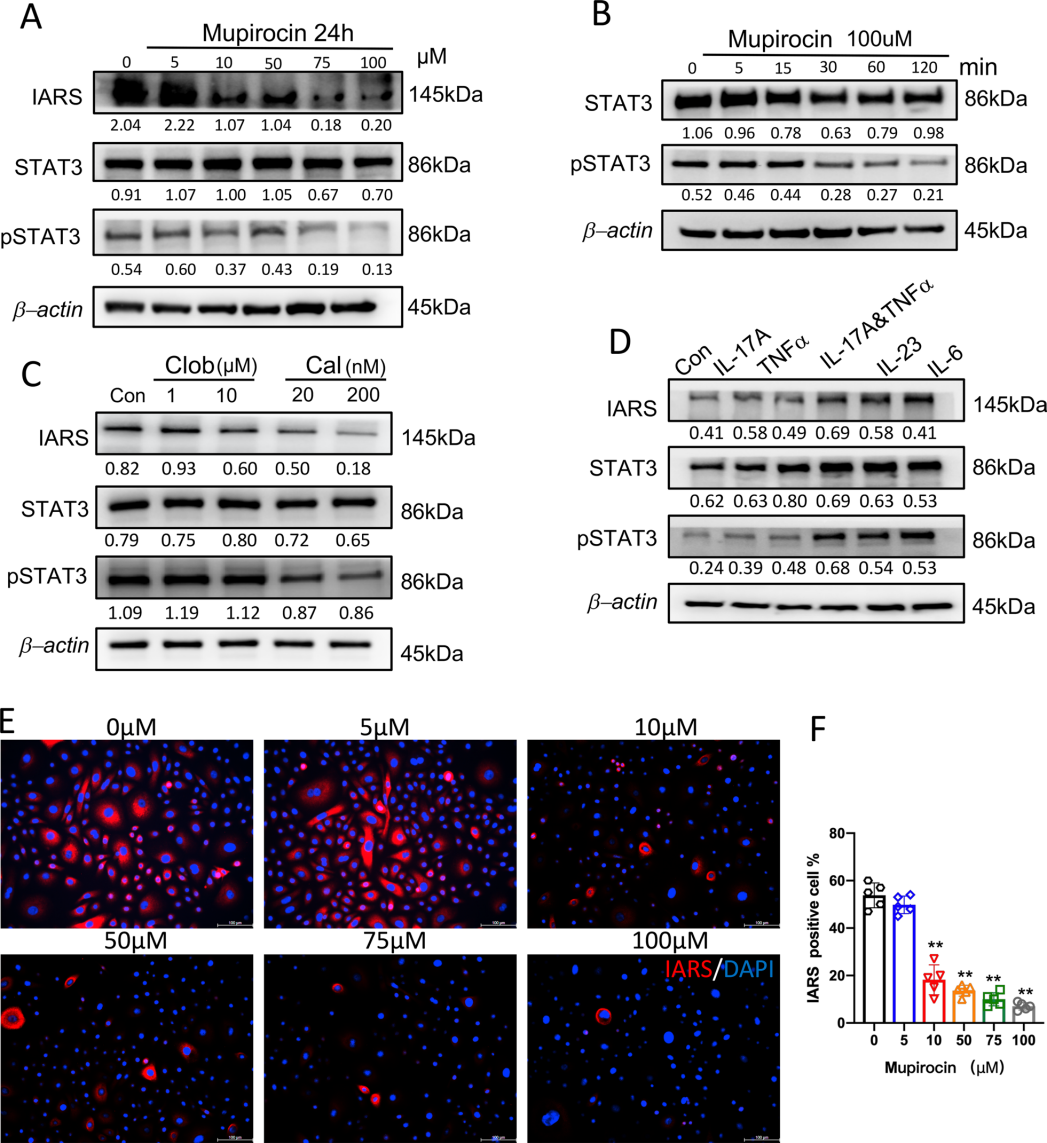
Regulation of IARS in PLEKs and NHEKs related to STAT3. **A** A Western blot analysis was performed to measure the expression of IARS, STAT3 and pSTAT3(Tyr 705) in PLEKs treated with various concentrations of mupirocin for 24 h. *β*-actin was used as loading control. **B** A Western blot analysis was performed to measure the expression of STAT3 and pSTAT3(Tyr 705) in PLEKs treated with 100 μM mupirocin for different minutes. **C** A Western blot analysis was performed to measure the expression of IARS, STAT3 and pSTAT3(Tyr 705) in PLEKs treated with clobetasol(1 and 10 μM) and calcipotriol (20 and 200 nM) for 48 h. **D** A Western blot analysis was performed to measure the expression of IARS, STAT3 and pSTAT3(Tyr 705) in NHEKs treated with IL-17A(200 ng/ml),TNFα(10 ng/ml),IL-17A(200 ng/ml) combine TNFα (10 ng/ml), IL-23(100 ng/ml) and IL-6(100 ng/ml) for 24 h. Representatives immunofluorescence images (**E**) and quantification analysis (**F**) of IARS in PLEKs treated with various concentrations of mupirocin for 24 h. Scale Bar = 100 μm. (n = 5 ± SEM). **P < 0.01 versus 0 μM

To investigate the regulatory mechanism of mupirocin on keratinocytes, PLEKs were treated with various concentrations of mupirocin (0–100 μM) for 24 h. As shown in Fig. 6A, the expression of IARS was dramatically inhibited in MUP concentration-dependent way. Among these groups, the IF results of IARS showed similar results in PLEKs (*P* < 0.01; Fig. 6E, F). Interestingly, phosphorylation of STAT3 was also inhibited when the concentration reached 75 μM (Fig. 6A). As a result, we continued to use high-concentration of MUP,100 μM, to treat PLEKs at different time point. The results of Western blot analysis showed that the expression of p-STAT3 was remarkably down-regulated from 30 min until 120 min (Fig. 6B). Thus, mupirocin was able to inhibit IARS in keratinocytes, maybe through STAT3 signaling pathway. In addition to the inhibitory effect of mupirocin, it is necessary to explore whether drugs including clobetasol and calcipotriol could have similar effects in IARS. Here, IARS expression was examined in PLEKs using Western blot following a 48 h treatment with clobetasol (Clob, 1 μM and 10 μM) and calcipotriol (Cal, 20 nM and 200 nM). After calcipotriol treatment, the expression of IARS was decreased in a dose-dependent manner, concomitant with a slight decrease in STAT3 phosphorylation levels (Fig. 6C). In contrast, we did not observe significant changes of IARS or p-STAT3 levels when treated with clobetasol (Fig. 6C). Moreover, IL-17A (200 ng/ml) combined with TNFα (10 ng/ml), IL-23 (100 ng/ml), and IL-6 (100 ng/ml) could promote IARS expression and STAT3 phosphorylation in NHEKs (Fig. 6D).

## Discussion

Psoriasis is a common chronic recurrent inflammatory systematic disease, which is associated with substantial disease burden and negative impact on patients’ quality of life [30, 31]. However, the cause of the disease has not yet been fully understood. In this study we discovered a possible mechanism in the pathogenesis of psoriasis. We found that IARS had a high level of protein in the psoriatic lesion. In addition, MUP was able decrease the expression of IARS and significantly inhibited IMQ-induced psoriasis like dermatitis by down-regulating IL-17 signaling pathway. Moreover, MUP induced apoptosis and inhibited the proliferation in PLEKs, which is related the STAT3 signaling pathway.

Previous research has identified the importance of disordered keratinocyte signaling and predisposition to type 17 responses that drive a pathogenic IL-17 loop in psoriasis [32, 33]. Similarly, we demonstrated that the process of MUP inhibiting IARS was located in the epidermis, especially in the keratinocytes (Fig. 1A). Besides, the RNA-sequencing analysis also showed the IL-17 pathway being involved (Fig. 3D). We indicated that MUP attenuated IMQ-induced psoriasis-like inflammation by reducing infiltrations of CD4^+^T cells, CD8^+^T cells, Ly6G^+^ neutrophils in the lymphoid nodes and increasing CD207^+^ LCs in the epidermis (Fig. 4). According to the keratinocytes and immune cell network in psoriasis lesional skin [34, 35], the skin related immune response should also be analyzed in a subsequent study.

The abnormal production of inflammatory cytokines, such as TNF-α, IL-6, IL-17 and IL-23, are confirmed to play key roles in psoriasis [36–38].The protein level of IARS was up-regulated in NHEKs treated with TNF-α, IL-6, IL-17 and IL-23(Fig. 6D). This implicates that MUP and IARS was associated with an immune environment in psoriasis. Besides, the underlying mechanism associated with STAT3 signaling pathway in keratinocytes treated with MUP has been identified (Fig. 6A-C).

Because STAT3 has emerged as a key role in the development and pathogenesis of psoriasis [39, 40], and K5.Stat3C mice has spontaneously developed psoriasis-like lesions [41, 42]. Therefore, STAT3 function in the MUP treatment needs to be further confirmed in vivo model.

In summary, MUP inhibits the formation of IARS, and subsequently 1.) decreases the expression of inflammatory cytokines and chemokines, and 2.) thereby reduces the infiltration of immune cells and 3.) alleviates IMQ-induced psoriasis-like dermatitis. MUP may inhibit the proliferation of psoriatic keratinocytes and promote their apoptosis by inhibiting the activation of STAT3 signaling pathway. Our research may provide another treatment alternation of psoriasis.

## Conclusions

In conclusion, this study shows that epidermal IARS is positively correlated with the activity of psoriasis inflammation. Mupirocin decreases inflammation through inhibiting epidermal IARS. Mupirocin inhibits the proliferation of PLEKs and promotes their apoptosis by inhibiting the activation of STAT3 signaling pathway.

## Supplementary Information

**Additional file 1:** Quantitative PCR primer sequences and flow cytometry antibodies.

**Additional file 2:** Flow cytometry gating strategy.

## Data Availability

The datasets used and/or analysed during the current study are available from the corresponding author on reasonable request.

## Abbreviations

Cal: Calcipotriol
CCL: Chemokine C–C motif ligand
Clob: Clobetasol
CXCL: Chemokine C–X–C motif ligand
DAPI: 4ʹ,6-Diamidino-2-phenylindole dihydrochloride
DC: Dendritic cell
ELISA: Enzyme linked immunosorbent assay
IARS: Isoleucyl-tRNA synthetase
IFN: Interferon
ILC: Innate lymphoid cell
IL-6: Interleukin-6
IL-12: Interleukin-12
IL-17: Interleukin-17
IL-23: Interleukin-23
IMQ: Imiquimod
KCs: Keratinocytes
KRT14: Keratin14
LCs: Langerhans cells
LCN2: Lipocalin-2
MMP-9: Matrix metalloproteinase-9
NHEKs: Normal human epidermal keratinocytes
PASI: Psoriasis area and severity index
PLEKs: Psoriatic lesional epidermal keratinocytes
qRT-PCR: Quantitative reverse transcription polymerase chain reaction
STAT3: Signal transduction and transcriptional activator3
Th: T helper cells
TLR: Toll like receptor
TNF-α: Tumor necrosis factor-α
Treg: Regulatory T cell
