# Supplementary material for: Mupirocin blocks imiquimod-induced psoriasis-like skin lesion by inhibiting epidermal isoleucyl-tRNA synthetase
Source: Cell Commun Signal. 2022 Nov 22;20:185. doi: 10.1186/s12964-022-00995-0 (PMC9682813; doi:10.1186/s12964-022-00995-0)
Supplement: Supplementary file 2 — Additional file 1: Quantitative PCR primer sequences and flow cytometry antibodies. [file 12964_2022_995_MOESM2_ESM.docx]

**Supplementary TABLE E1.** Quantitative PCR primer sequences

| Name | Orientation | Sequence 5’ 3’ |
| --- | --- | --- |
| *IARS* | fw | GCTTACCTGTGGAATATGAA |
|  | rev | GGCACTGATTGTTATACTCT |
| *β-Actin* | fw | CCTTCTTGGGTATGGAATC |
|  | rev | TGTTGGCATAGAGGTCTT |

*fw*, Forward; *rev*, reverse.

**Supplementary TABLE E2.** Flow cytometry antibodies

| Antibody(anti-) | Clone | Company | Concentration |
| --- | --- | --- | --- |
| CD3 | 17A2 | BioLegend | 1:100 |
| CD4 | GK1.5 | BioLegend | 1:100 |
| CD8a | 53-6.7 | BioLegend | 1:100 |
| CD11c | N418 | BioLegend | 1:100 |
| CD45 | 30-F11 | BioLegend | 1:100 |
| CD207 | 4C7 | BioLegend | 1:100 |
| F4/80 | BM8 | eBioscience | 1:100 |
| IL-17A | eBio17B7 | eBioscience | 1:100 |
| Ly-6G | 1A8 | BioLegend | 1:100 |
| MHCII | M5/114.15.2 | BioLegend | 1:100 |
| TCRγδ | GL3 | BD Biosciences | 1:100 |

Beckman Coulter (Brea, Calif); BioLegend (San Diego, Calif); eBioscience (Thermo Fisher Scientific).
