# Supplementary material for: Mupirocin blocks imiquimod-induced psoriasis-like skin lesion by inhibiting epidermal isoleucyl-tRNA synthetase
Source: Cell Commun Signal. 2022 Nov 22;20:185. doi: 10.1186/s12964-022-00995-0 (PMC9682813; doi:10.1186/s12964-022-00995-0)
Supplement: Supplementary file 3 — Additional file 2: Flow cytometry gating strategy. [file 12964_2022_995_MOESM3_ESM.docx]

**
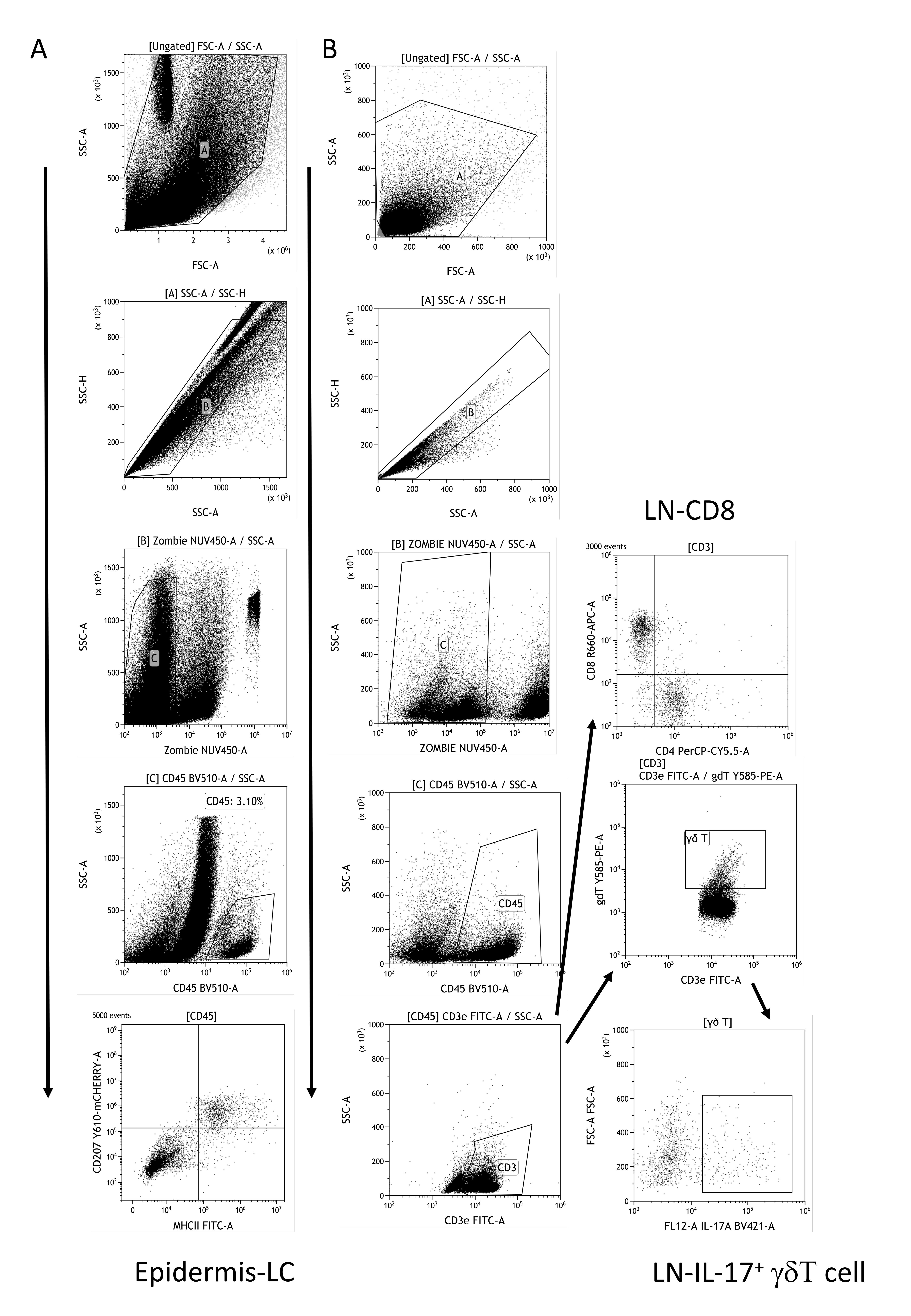
**

**
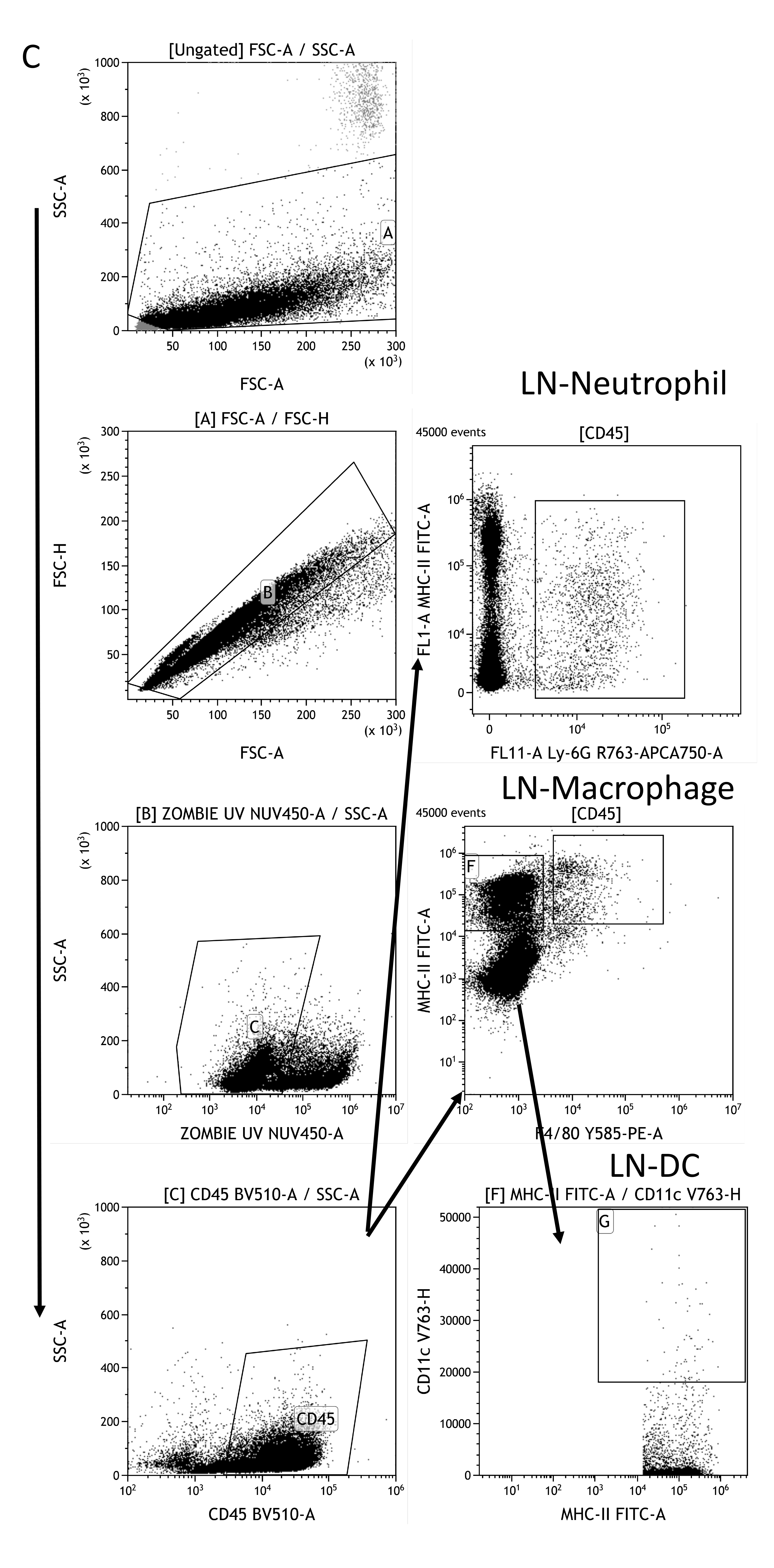
**

**Supplementary Figure s1** Mupirocin altered immunocytes compositions in IMQ-treated mice. (a) Flow cytometry gating strategy used to identify epidermal LCs (live CD45^+^MHCII^+^CD207^+^) in single-cells suspensions isolated from the epidermis of mice. (b) Flow cytometry gating strategy used to identify lymph node CD8^+^TCs (live CD45^+^CD3^+^CD8^+^) and IL-17^+^ γδT cell (live CD45^+^ CD3^+^TCRγδ^+^IL-17A^+^) in single-cells suspensions isolated from the mice. (c) Flow cytometry gating strategy used to identify lymph node neutrophils (live CD45^+^Ly6G^+^), macrophage(live CD45^+^MHCII^+^F4/80^+^) and DC(live CD45^+^MHCII^+^F4/80^-^CD11c^+^) cell in single-cells suspensions isolated from the mice. FSC-A, forward scatter area; FSC-H, forward scatter height; LC, Langerhans cell; SCC-A, side scatter area; DC, Dendritic cell.
